# HPA axis dysregulation is associated with differential methylation of CpG-sites in related genes

**DOI:** 10.1038/s41598-021-99714-x

**Published:** 2021-10-11

**Authors:** Andreas Chatzittofis, Adrian Desai E. Boström, Diana M. Ciuculete, Katarina Görts Öberg, Stefan Arver, Helgi B. Schiöth, Jussi Jokinen

**Affiliations:** 1 Medical School, University of Cyprus, 1678 Nicosia, Cyprus; 2 Department of Clinical Sciences/Psychiatry, Umeå University, Umeå, Sweden; 3 Neuropaediatric Unit, Department of Women’s and Children’s Health, Karolinska Institutet, Stockholm, Sweden; 4 Department of Neuroscience, Uppsala University, Uppsala, Sweden; 5 Department of Medicine, Karolinska Institute, Karolinska University Hospital, Stockholm, Sweden; 6 Institute for Translational Medicine and Biotechnology, Sechenov First Moscow State Medical University, Moscow, Russia; 7 Department of Clinical Neuroscience/Psychiatry, Karolinska Institutet, Stockholm, Sweden

**Keywords:** Genetics, DNA methylation, Epigenetics in the nervous system

## Abstract

DNA methylation shifts in Hypothalamic–pituitary–adrenal (HPA) axis related genes is reported in psychiatric disorders including hypersexual disorder. This study, comprising 20 dexamethasone suppression test (DST) non-suppressors and 73 controls, examined the association between the HPA axis dysregulation, shifts in DNA methylation of HPA axis related genes and importantly, gene expression. Individuals with cortisol level ≥ 138 nmol/l, after the low dose (0.5 mg) dexamethasone suppression test (DST) were classified as non-suppressors. Genome-wide methylation pattern, measured in whole blood using the EPIC BeadChip, investigated CpG sites located within 2000 bp of the transcriptional start site of key HPA axis genes, i.e.: *CRH*, *CRHBP*, *CRHR-1*, *CRHR-2*, *FKBP5* and *NR3C1*. Regression models including DNA methylation M-values and the binary outcome (DST non-suppression status) were performed. Gene transcripts with an abundance of differentially methylated CpG sites were identified with binomial tests. Pearson correlations and robust linear regressions were performed between CpG methylation and gene expression in two independent cohorts. Six of 76 CpG sites were significantly hypermethylated in DST non-suppressors (nominal *P* < 0.05), associated with genes *CRH*, *CRHR1*, *CRHR2*, *FKBP5* and *NR3C1*. *NR3C1* transcript AJ877169 showed statistically significant abundance of probes differentially methylated by DST non-suppression status and correlated with DST cortisol levels. Further, methylation levels of cg07733851 and cg27122725 were positively correlated with gene expression levels of the NR3C1 gene. Methylation levels of cg08636224 (*FKBP5)* correlated with baseline cortisol and gene expression. Our findings revealed that DNA methylation shifts are involved in the altered mechanism of the HPA axis suggesting that new epigenetic targets should be considered behind psychiatric disorders.

## Introduction

The hypothalamic–pituitary–adrenal (HPA) axis is a key regulator of the response to stress and is involved in the adaptation and homeostasis of the organism under a variety of circumstances^1^. In the recent decades HPA axis dysregulation has been reported in various psychiatric disorders including, but not limited to, major depressive disorder^2^, post-traumatic stress disorder (PTSD)^3^, addiction^4^ and hypersexual disorder^5^. One possible explanation to the association seen between HPA axis dysfunction and psychiatric morbidity could be through epigenetic mechanisms.

Epigenetics, and specifically DNA-methylation is recognized as the most promising potential pathway in mediating the nature-nurture interactions. DNA methylation results in a reversible structural change which subsequently has effects on gene expression mainly through altered binding of transcription factors^6^. Although previously thought that DNA-methylation essentially always resulted in silencing of affected genes, it now seems that the exact genomic sites of methylation determine what the downstream consequences are for gene transcription^6,7^. Therefore, it is very important to elucidate the exact effects of gene methylation on specific genomic sites.

Preliminary studies on differential methylation of genes involved in the HPA axis function have been mainly focused on the glucocorticoid-receptor encoding gene (*NR3C1*) and the FKBP Prolyl Isomerase 5 gene (*FKBP5*). Although there are exceptions, the majority of the studies report lower *NR3C1* methylation in PTSD, while results remain mixed in depression^8,9^. Findings are very preliminary in other psychiatric disorders such as hypersexual disorder^10^. Moreover, it is imperative to point out that previous studies have mainly focused only on specific genes, not including gene expression analyses, or cortisol levels and other measurements of the HPA axis function^8^. Consequently, even though some studies have found associations between psychiatric disorders, HPA axis dysfunction and DNA-methylation changes, what is hitherto still largely unknown is in what way exactly differential methylation of which CpG-sites in what genes is associated with HPA axis dysregulation. In addition, cortisol is reported to have an inhibitory effect on the HPT (hypothalamic pituitary-thyroid) axis although studies report mixed results^11–13^.

The aim of this study was to examine the association between HPA-axis dysregulation measured with the dexamethasone suppression test (DST) and differential DNA-methylation of key HPA axis related genes in a study population comprising patients with hypersexual disorder and healthy volunteers. Moreover, we assessed the relation between DNA methylation of the HPA axis related genes and the function of the thyroid gland. Finally, we aimed to examine possible association of these methylation profiles to gene expression levels of selected genes using data from two independent cohorts.

## Materials and methods

### Ethics and patient consent

The study protocols were approved by the Regional Ethical Review Board in Stockholm (Dnrs: 00–194, 2015/1454–32) and the participants gave their written informed consent to the study. Concerning the independent cohort and expression data set, both studies were approved by the Regional Ethical Review Board in Uppsala, and all participants gave their written informed consent. All methods were carried out in accordance with relevant guidelines and regulations.

### Characterization of the discovery group

The study population has been described previously in detail^5,10^. Sixty-seven hypersexual patients were recruited through advertising in media as well as referrals at the Center for Andrology and Sexual Medicine (ANOVA) at the Karolinska University Hospital. A diagnosis of hypersexual disorder, age of 18 years or older and available contact information were the inclusion criteria.

A current psychotic illness, current alcohol/drug abuse or serious physical illness and psychiatric disease requiring immediate treatment were the exclusion criteria. The diagnosis of hypersexual disorder was set by a trained psychiatrist and psychologist according to proposed DSM 5 criteria (participants needed 4 out of 5 criteria to be included)^14^. The Mini International Neuropsychiatric Interview (MINI) was used for other psychiatric diagnoses^15^.

Healthy male volunteers were recruited from the Karolinska Trial Alliance (KTA) database and after screening, were included if they had no previous or current psychiatric illness, no first degree relative with completed suicide, bipolar disorder, or schizophrenia; and no previous exposure to serious trauma (natural disaster, assault) and no serious physical illness. Also, individuals screened positive for HD or pedophilic disorder were also excluded. The healthy volunteers were age match with HD patients, and the time of blood sampling was matched to either fall or spring to minimize potential seasonal variations. One volunteer of the total 40 was excluded due to physical illness detected in the laboratory results leaving a sample of 39 healthy volunteers.

### Assessments and clinical data of the discovery group

All participants (HD patients and healthy volunteers) were assessed using the Mini-International Neuropsychiatric Interview (MINI 6.0) (Sheehan et al., 1998), the Hypersexual Disorder Screening Inventory (HDSI) (www.dsm5.org), the Hypersexual Disorder: Current Assesssment Scale (HD:CAS), the Sexual Compulsivity Scale (SCS)^16^, the Montgomery-Åsberg Depression Rating Scale Self-rating (MADRS-S)^17^ and the Childhood Trauma Questionnaire (CTQ)^18^. For more details please see supplementary material.

### Blood samples and analysis

Blood samples from non-fasting participants were collected in the morning according to standard procedures. Analyses of plasma ACTH and Cortisol assays were performed directly after sampling at the laboratory of the Karolinska University Hospital using a chemiluminescence immunoassay. To test the HPA axis function, low dose (0.5 mg at 23:00 h) dexamethasone suppression test (DST) was performed in all participants the same day after the baseline plasma samples of ACTH and Cortisol were gathered. Post-DST blood samples were collected the next day at approximately 08.00 h and analyzed using the same method as for baseline ACTH and Cortisol. Individuals having plasma Cortisol level of 138 nmol/l (equivalent to = 5 g/dl) or higher in the morning sample after dexamethasone administration was classified as non-suppression (impaired feedback inhibition).

### Blood sample collection and methylation profiling

Genomic DNA was extracted from 110 samples using the phenol–chloroform method^19^. Subsequently, the EZ DNA Methylation—GoldTM kit (ZymoResearch, USA) was used for bisulfite conversion. Bisulfite converted DNA was thereafter hybridized to the Illumina Infinium Methylation EPIC BeadChip, measuring the methylation state of over 850-K CpG sites. The array was imaged in using the Illumina iScan system (Illumina, San Diego, CA, USA) in which the percent methylation state of each CpG site was quantified across the study group.

### Data processing

Preprocessing of the methylation data was performed by background correction, adjustment of probe type differences, removal of batch effects and probe exclusion. Subsequently, the global DNA methylation pattern was adjusted for white blood cell type heterogeneity. Principal component analysis (PCA) was used to identify sample outliers in the methylation data. Methylation preprocessing steps were performed in using the minfi^20^, watermelon^21^, sva^22^, ChAMP^23^ and FactoMineR^24^ packages of the Bioconductor project operable in R, version 3.3.0. For background correction, adjustment of type I and type II probes, removal of batch effects and probe exclusion please see supplementary material.

### Criteria of sample exclusion

To investigate the global DNA methylation pattern for sample outliers, the ‘PCA’ function of the FactoMineR package was used^24^. 7,547 probes were further studied and included in the covariance matrix based on a threshold of 0.2 and a 95% reference range, as performed by Voisin et al.^25^. The first principal component explained 20.4% of the total variance and successively studied vectors did not add significantly to the total variance. Outliers were identified by visual inspection of the graphical display of the first principal component, resulting in seventeen samples being excluded from further analysis.

### CpG site annotation and selection of HPA axis coupled probes

90% of the probes on the Illumina EPIC BeadChip array are also present on the Illumina 450 K Methylation Beadchip. We therefore used the expanded annotation produced by Price et al., originally designed for the 450 K array, to define, for each CpG site, the distance to the closest transcriptional start site (TSS) and the associated gene^26^. As such, only CpG-sites present on the Illumina 450-K methylation beadchip were considered for further analysis. In addition, we only considered CpG sites located within 2,000 base pairs (bp) up and downstream of the TSS. Wagner et al. demonstrated that DNA methylation and gene expression is closely related in this region^7^.

We considered the following HPA axis coupled genes: *CRH*, corticotropin releasing hormone binding protein (*CRHBP*), corticotropin releasing hormone receptor 1 (*CRHR1*), corticotropin releasing hormone receptor 2 (*CRHR2*), *FKBP5* and the *NR3C1*. After the preprocessing steps outlined above, 76 CpG sites annotated to any of the aforementioned genes were investigated in the subsequent analysis.

### Methylation-expression correlation

Candidate CpG sites were investigated for a correlation between methylation and expression in two independent cohorts.

#### FTO cohort

11 healthy male volunteers aged between 18 and 40 years were recruited from the region of Uppsala, Sweden, between 2013 and 2014. Blood analyses were performed before and after a meal intake. For the purpose of this study, only the non-fasting blood samples were further studied. The genome-wide DNA methylation pattern was measured using the Illumina Infinium 450 K BeadChip. RNA microarray expression was measured and analyzed using the Affymetrix GeneChip Human Gene 2.1 ST array. More details on the cohort and preprocessing of the methylation and RNA specimens have been previously published^27^.

#### E-GEOD-49065 cohort

Data is openly available (E-GEOD-49065) and were originally published by Steegenga et al., who studied age-induced changes in DNA methylation and their effect on gene expression. Methylation data was measured using the Illumina Infinium 450 K BeadChip and expression data with the Affymetrix Human Gene 1.1. ST array. The cohort of methylation and expression data in peripheral blood mononuclear cells (PBMCs) included 10 healthy Caucasian male blood donors, aged 30–66 years^28^.

### Statistical analysis

All statistical analyses were performed in using R statistics, version 3.3.0.

After the preprocessing steps, 76 HPA axis coupled CpG sites and 93 samples remained to be included in the subsequent analysis. Skewness and kurtosis of the distribution of continuous variables were evaluated with the Shapiro-Wilks test. Baseline cortisol levels and HbA1C (mml/mol) were normally distributed in both hypersexuality patients and in healthy volunteers, whereas the other clinical variables were not. The t-test and Kruskal–Wallis’ test were subsequently used to investigate group differences in continuous variables between hypersexuality patients and healthy controls. Chi-squared tests were used to detect differences in categorical variables, e.g. gender, depression and DST non-suppression status.

There were many potential covariates on the association analysis between DNA methylation and DST non-suppression status, e.g. hypersexuality, depression, CTQ Total, TSH/T4-quota, HbA1C, baseline cortisol and ACTH, and plasma levels of testosterone, TNF-alpha and IL-6. To avoid overfitting by including too many covariates, we investigated each individual covariate against the phenotype of interest in multiple linear regression models using the ‘lm’ function in R. Covariates were incrementally and independently selected. Using the computed analysis of variance, we tested whether the addition of a particular covariate resulted in a better fit to the model and only included variables with a p-value < 0.10. The best linear model for DST non-suppression status included the CpG sites, hypersexuality (*P* = 0.033) and baseline cortisol (*P* = 0.087). We considered the same co-variates on the association analyses between DNA methylation and baseline cortisol and ACTH, DST cortisol, DST ACTH and the TSH/T4-ratio. The optimal model for cortisol and ACTH, at baseline and after DST, included only the CpG-sites. For the TSH/T4-ratio, the best linear model included the methylation sites, hypersexuality (*P* = 0.066) and IL-6 (*P* < 0.001).

Beta-values of methylation were used for graphical illustration. For statistical analysis, we transformed the beta values to M-values, which have been shown to be statistically more robust^29^.The association analysis between DNA methylation and the phenotypes of interest was tested with linear models using the ‘limma’ package for R, applying an empirical Bayes method based on a moderated t-statistic^30^. We assumed a linear model where the M values of each CpG site were used as a quantitative dependent trait and the phenotypes of interest were used as covariates together with the optimal covariates identified in the analysis outlined above. All analyses were accounted for multiple testing using the false discovery rate (FDR) method^31^. As a second step, as each gene transcript may be associated with several CpG sites, we analyzed the results of the regression analyses to identify gene transcripts with an abundance of differentially methylated CpG sites using binomial tests. P-value thresholds (‘hypothesized probability of success’) were set to 0.05 to stratify probes according to significant and non-significant methylation changes. Binomial tests were then performed in R using the function “binom.test”, contrasting for each gene the number of nominally significant CpG sites to the total number of probes annotated to each gene transcript not taking the direction of the methylation change into account. Binomial test p-values were adjusted for multiple testing using the Bonferroni method. Gene transcripts with a Bonferroni-adjusted binomial test *P*-value < 0.05 were considered significant.

Candidate CpG sites were further investigated with regard to their association with transcriptional expression of the respective gene in two independent cohorts (FTO cohort and E-GEOD-49065 cohort). Methylation M-values were correlated with normalized gene expression data in two separate regressions, using both Pearson’s product moment correlation method and robust linear regressions using the ‘lmRob’ function of the “robust” package for R^32^. On the analysis of TSH/T4 associated CpG sites, in the case of more than one candidate methylation probe for each gene transcript, methylation levels were averaged for each gene. Correlation analyses between methylation and the level of transcriptional expression were in these cases performed in using averaged methylation values. Finally, post-hoc analyses of candidate CpG-sites to investigate the effect of hypersexuality on methylation were performed as well as blood–brain correlations of the candidate CpG-sites using the Blood–Brain Epigenetic Concordance (BECon) tool^33^.

## Results

### Behavior of the clinical outcome variables

In this cohort of 20 DST non-suppressors and 73 DST suppressors (controls), DST non-suppressors had significantly higher levels of DST cortisol (*P* < 0.00001) and DST ACTH (*P* < 0.0001). In addition, DST non-suppressors tended to suffer from hypersexuality to a larger degree than controls (*P* = 0.0578). No between group differences were identified for age, gender, CTQ score, TSH/T4-quota, baseline cortisol, baseline ACTH, plasma levels of testosterone, TNF-alpha or IL-6 (Table 1).

**Table 1:** Clinical characteristics of DST non-suppressors and controls.

	DST non-supressors	Controls	Statistics (t-test, Kruskall-Wallis, Chisq.test), *P* value
N	20	73	
Age (years)	37.4 (12.1)	39 (11.6)	ns
Men:Women, (n (%))	18 (90) : 2 (10)	69 (5.5) : 4 (5.5)	ns
Diagnosis depression (n(%))	3 (15.0)	6 (8.2)	ns
Hypersexuality (n(%))*	17 (85.0)	43 (58.9)	5.78E-02
CTQ Total	37.2 (13.4)	37.8 (11.2)	ns
TSH (mE/L)/T4 (nmol/L)	0.017 (0.0099)	0.023 (0.02333)	ns
HBA1C (mml/mol)	31.7 (2.9)	33.2 (5.5)	ns
Cortisol (nmol/L)	515.2 (147.2)	458.5 (133.5)	ns
DST Cortisol (nmol/L)	225.8 (92.5)	50.6 (34.2)	**5.74E−11**
ACTH (pmol/L)	5.94 (2.32)	6.23 (3.19)	ns
DST ACTH (pmol/L)	3.17 (1.79)	1.45 (1.08)	**3.37E−05**
Testosteron (nmol/L)	13.9 (4.1)	14.1 (5.7)	ns
TNF-alpha (ng/L)	6.9 (2.1)	6.7 (2.2)	ns
IL-6 (ng/L)	2.03 (0.12)	2.30 (0.98)	ns

Values are shown as mean (SD) unless otherwise specified. P-values were calculated by means of t-test, Kruskall-Wallis’ test or chi-squared tests, contrasting values for DST non-suppressors and controls (DST suppressors). A one-tailed p-value < 0.05 was considered significant.

*CTQ* childhood trauma questionnaire, *DST ACTH* ACTH levels after the dexamethasone suppression test, *DST Cortisol* cortisol levels after the dexamethasone suppression test; DST non-suppressors, non-suppression status defined as DST cortisol levels ≥ 138 nmol/l, *ns* not significant.

### Investigation of 76 HPA axis coupled probes reveals *NR3C1* transcript AJ877169 to have a statistically significant abundance of probes differentially methylated by DST non-suppression status

We performed multiple linear regression models of methylation M-values to a binary outcome variable of DST non-suppression status, adjusting for hypersexuality and baseline cortisol levels. 76 individual CpG sites were tested, and six of these were by nominal significance hypermethylated in DST non-suppressors (*P* < 0.05), associated with the genes *CRH, CRHR1, CRHR2, FKBP5* and *NR3C1*. No individual CpG site was significant after corrections were made for multiple testing using the FDR-method (please see supplementary material). With two out of two CpG sites significantly hypermethylated in DST non-suppressors (cg07733851, cg27122725; *P* < 0.05), *NR3C1* associated transcript AJ877169 was the only gene transcript with a statistically significant abundance of differentially methylated CpG sites after Bonferroni corrections were made for multiple testing (please see supplementary material).

### AJ877169 is shown to have also a statistically significant abundance of CpG sites correlated with DST cortisol levels

The analysis performed between the 76 HPA axis coupled CpG sites and plasma levels of DST cortisol revealed the same *NR3C1* (AJ877169) associated CpG sites identified in the analysis of DST non-suppression status—cg07733851 and cg27122725—were nominally significant (*P* < 0.05). Additional, probes associated with the *CRH* and *FKBP5* genes were identified. No individual CpG site was significant after adjustments for multiple testing (please see supplementary material). AJ877169 had a statistically significant abundance of differentially methylated CpG sites after Bonferroni adjustments (please see supplementary material).

### Methylation levels of cg07733851 and cg27122725 are positively correlated with gene expression levels of the *NR3C1* gene

To evaluate to what extent the methylation of candidate CpG sites associated with DST non-suppression status and plasma DST cortisol levels impacts the expression of *NR3C1*, Pearson’s correlation analyses and robust linear regression analyses were performed in two separate cohorts of healthy controls (FTO cohort, n = 11; E-GEOD-49065, n = 10, respectively). The methylation state and level of transcriptional expression was aligned to each other intra-individually, not taking any covariates into account. We found a significant positive correlation with transcription for cg27122725 in the robust model of the E-GED-49065 cohort (*P* < 0.05). In addition, cg07733851 was positively correlated with *NR3C1* expression levels in the robust model of the FTO cohort (*P* < 0.001), (Table 2).

**Table 2:** Methylation/transcription correlations of HPA-axis associated CpG sites differentially methylated by DST non-suppression status and positively correlated with DST cortisol levels.

Gene	Transcript	Illumina ID	FTO-cohort (n = 11)				E-GEOD-49065 (n = 10)			
			Robust linear regression		Pearson correlation analyses		Robust linear regression		Pearson correlation analyses	
			Coef	*P*.val	Coef	*P*.val	Coef	*P*.val	Coef	*P*.val
*NR3C1*	AJ877169	cg07733851	**0.53**	**6.56E-04**	–	*ns*	–	*ns*	–	*ns*
*NR3C1*	AJ877169	cg27122725	–	*ns*	–	*ns*	**0.47**	**4.04E-02**	–	*ns*

The table lists CpG-sites located within the TSS2000 of HPA-axis genes with significant cortisol post DST-associated methylation changes. These methylation probes are investigated for a correlation with transcription in two separate cohorts with healthy controls (FTO-cohort and E-GEOD-49065 cohort). Methylation M-values were correlated with expression values inter-individually, by pearson correlations and robust linear regression models, respectively.

*Coef*. regression coefficient; *P*.val, *P*-value.

### No HPA axis coupled transcript or individual CpG site was significantly correlated with ACTH levels, both pre and post DST

In the association analyses between DNA methylation and ACTH levels pre and post DST, no individual CpG site was significant after adjustments were made for multiple testing (please see supplementary material). Furthermore, there were no gene transcripts with an abundance of nominally significant probes (data not shown).

### Methylation levels of cg08636224—associated with ***FKBP5*** transcript NM_004117—is significantly correlated with baseline cortisol

**Figure 1:**
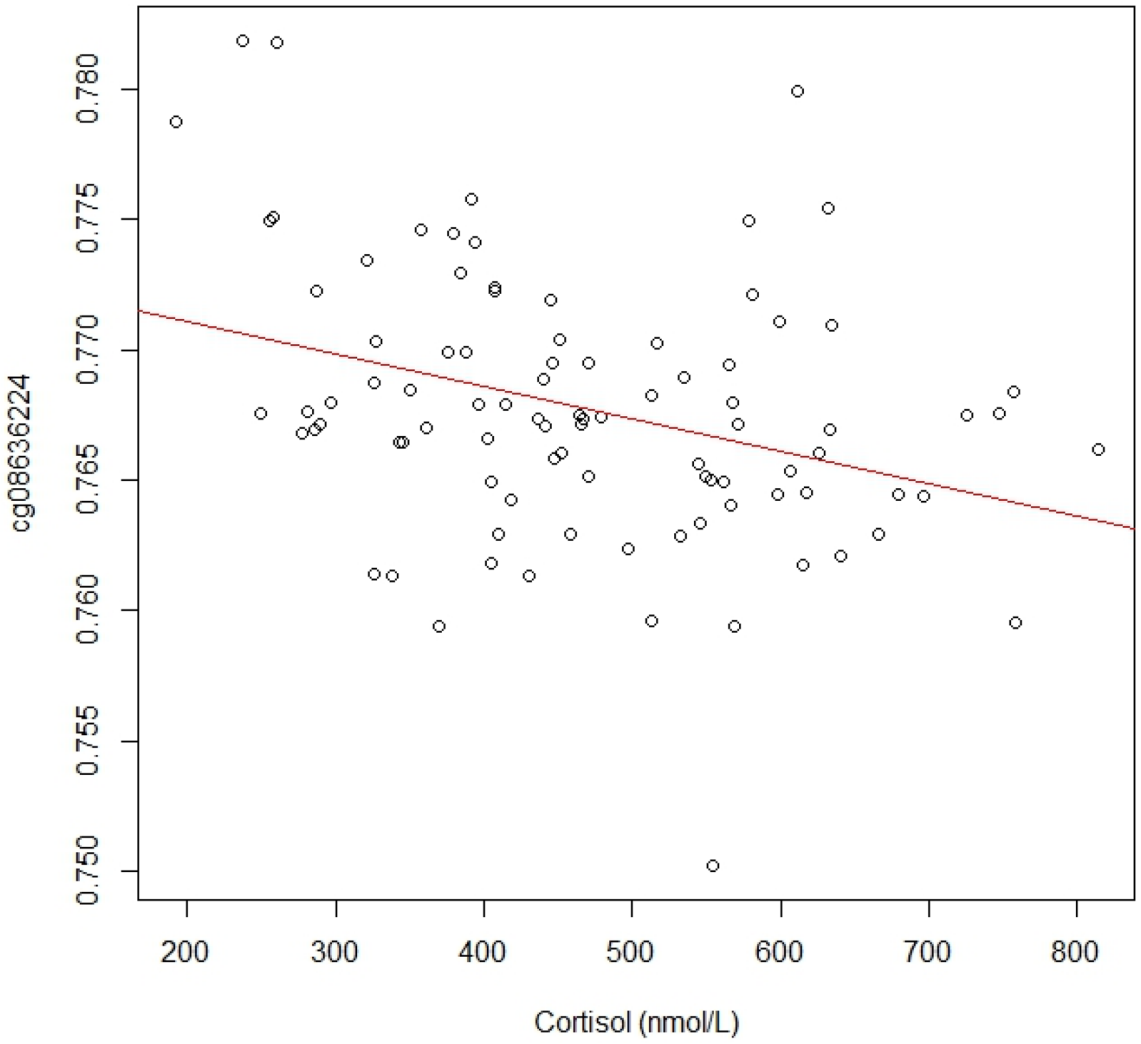
Scatterplot of FKBP5 associated CpG site cg08636224 methylation levels and baseline cortisol (nmol/L).

We also investigated associations of baseline cortisol and HPA axis coupled methylation probes. In this analysis, DNA-methylation levels at three CpG sites were inversely correlated with the baseline cortisol (*P* < 0.05). The identified CpG sites were, cg23185751(*CRHR2*), cg08636224(*FKBP5*) and cg07733851(*NR3C1*). After adjustments were made for multiple-testing, methylation levels of cg08636224, associated with the *FKBP5* gene and the NM_004117 transcript, was significant (pFDR = 0.0269)(please see supplementary material), (Fig. 1).

### Methylation levels of cg08636224—significantly associated with baseline cortisol—is positively correlated with gene expression of the FKBP5 gene

To investigate the impact of candidate CpG site cg08636224 on transcriptional activity of the associated gene, the methylation state was correlated with the level of transcriptional expression of the *FKBP5* gene intra-individually. In the E-GEOD-49065 cohort, we found significant positive correlations with gene expression in both robust and Pearson correlation analyses. This finding was, however, not replicated in the FTO-cohort (Table 3).

**Table 3:** Methylation/transcription correlations of FKBP5 associated CpG site cg08636224 which is negatively correlated with baseline cortisol levels.

Gene	Transcript	Illumina ID	FTO-cohort (n = 11)				E-GEOD-49065 (n = 10)			
			Robust linear regression		Pearson correlation analyses		Robust linear regression		Pearson correlation analyses	
			Coef	*P*.val	Coef	*P*.val	Coef	*P*.val	Coef	*P*.val
*FKBP5*	NM_004117	cg08636224	-	*ns*	**-**	*ns*	**0.84**	**2.18E−02**	**0.74**	**1.49E−02**

The table lists CpG-sites located within the TSS2000 of HPA-axis genes with significant cortisol post DST-associated methylation changes. These methylation probes are investigated for a correlation with transcription in two separate cohorts with healthy controls (FTO-cohort and E-GEOD-49065 cohort). Methylation M-values were correlated with expression values inter-individually, by pearson correlations and robust linear regression models, respectively.

*Coef*. regression coefficient; *P*.val, *P*-value.

### CpG sites associated with the genes *CRH, CRHR1, CRHR2* and *FKBP5* are significantly correlated with the TSH/T4-ratio

We investigated associations between DNA methylation and the TSH/T4-ratio by multiple linear regressions of methylation M-values to the phenotype of interest, adjusting for hypersexuality disorder and plasma levels of IL-6. After adjustments for multiple testing, nine methylation probes were significantly correlated with the TSH/T4-ratio, associated with the genes *CRH, CRHBP, CRHR1, CRHR2* and *FKBP5* (please see supplementary material). Furthermore, with five out of 14 CpG sites nominally significant (*P* < 0.05), *CRHR2* associated transcript EU012442 had a statistically significant abundance of CpG sites that were correlated with the TSH/T4-ratio after the Bonferroni adjustments (please see supplementary material).

### Candidate methylation sites correlated with the TSH/T4-ratio and associated with the genes *CRH* and *CRHBP* are significantly correlated with gene expression levels

As there were more than one candidate CpG sites for *CRHR1* transcript EU012435 and *CRHR2* transcript EU012442, we used averaged methylation values on the association analysis with levels of transcriptional expression. Methylation levels of *CRHBP* associated probe cg21842274 was significantly negatively correlated with gene expression levels in the Pearson analyses in both the FTO and E-GEOD-49065 cohort (*P* < 0.05). In addition, CRH associated CpG site cg18640030 was positively correlated with gene expression levels in the robust model of the E-GEOD-49065 cohort (*P* < 0.01). No associations with gene expression levels were found for the CpG sites associated with *CRHR1, CRHR2* and *FKBP5* (Table 4).

**Table 4:** Methylation/transcription correlations of CpG sites correlated with TSH/T4 (mE/nmol).

Gene	Transcript	sign.^a^ CpG-sites	FTO-cohort (n = 11)				E-GEOD-49065 (n = 10)			
			Robust linear regression		Pearson correlation analyses		Robust linear regression		Pearson correlation analyses	
			Coef	*P*.val	Coef	*P*.val	Coef	*P*.val	Coef	*P*.val
*CRH*	NM_000756	1	-	*ns*	–	*ns*	**5.69**	**2.61E–03**	0.54	0.11
*CRHBP*	NM_001882	1	-	*ns*	**− 0.53**	**4.84E–02**	–	*ns*	**− 0.55**	**4.85E–02**
*CRHR1*	EU012435	2	-	*ns*	–	*ns*	–	ns	–	ns
*CRHR2*	EU012442	4	-	*ns*	–	*ns*	–	ns	–	ns
*FKBP5*	NM_004117	1	-	*ns*	–	*ns*	–	*ns*	–	*ns*

The table lists CpG-sites located within the TSS2000 of HPA-axis genes that are significantly correlated with TSH/T4-levels. Methylation probes belonging to the same transcript were first averaged and then investigated for a correlation with transcription in two separate cohorts of healthy controls (FTO-cohort and E-GEOD-49065 cohort). Methylation M-values were correlated with expression values inter-individually, by pearson correlations (assuming an inverse correlation) and robust linear regression models, respectively.

*Coef*. regression coefficient; *P*.val, *P*-value.

### Post-hoc analyses of candidate CpG-sites to investigate the effect of hypersexuality on methylation

For the DST non-suppression status, pre- and post-DST cortisol level analysis, multiple linear regressions of the CpG-site methylation levels to the dichotomous variables CpG ~ DST ns + hypersexual disorder + DST ns*hypersexual disorder we performed. DST non-suppression status was also investigated for interaction effects by binomial logistic regression models, DST ns ~ CG + hypersexual disorder + CG*hypersexual disorder. NR3C1-associated methylation site cg07733851 was significantly (*P* < 0.05) associated with post-DST cortisol levels and hypersexual disorder. Specifically, the interaction term composed of DST non-suppression status and occurrence of hypersexual disorder diagnosis had a positive coefficient (*P* < 0.05), indicating disease state-dependent effects on the association between methylation status and expression levels.

### Blood–brain correlations of the candidate CpG-sites using the BECon tool

Transferability between blood- and brain methylation levels occur specifically in the BA20 region of the brain for *NR3C1*-associated CpG-sites cg07733851 and cg27122725. In the case of cg27122725, the BA7-region appear of some functional relevance as well. (Please see supplementary material).

## Discussion

Our findings of epigenetic changes of the HPA axis associated genes being associated with HPA function, in the form of pre- and post DST cortisol output, as well as gene expression in independent cohorts, adds to the previous findings of the significance of epigenetic changes of HPA axis on a molecular level. Key elements of the HPA axis regulation include the glucocorticoid receptor, encoded by the *NR3C1* gene and other regulating factors such as the FK506 encoded by the *FKBP5* gene that regulates the activity of the glucocorticoid receptor. In this cohort, comprising 20 DST non-suppressors and 73 DST suppressors as controls, we initially aimed to identify HPA axis coupled CpG-sites, in which modifications of the epigenetic profile are associated with DST non-suppression status. DNA methylation at six CpGs (related genes *CRH, CRHR1, CRHR2, FKBP5* and *NR3C1*), were by nominal significance hypermethylated in DST non-suppressors and the *NR3C1* associated transcript AJ877169 was the only gene transcript with a statistically significant abundance of differentially methylated CpG sites after corrections for multiple testing. The same transcript was correlated with DST cortisol plasma levels. Moreover, methylation levels of CpG sites of the *NR3C1* gene (cg07733851 and cg27122725) were positively correlated with gene expression levels of the *NR3C1* gene in two independent cohorts. Furthermore, according to DNA methylation annotation by Illumina and Price et al.,^26^ none of the included CpG-sites in the analyses, were positioned in enhancers or activated enhancers. The AJ877169 associated candidate CpG-sites were both annotated as promoter associated. Both studied candidate CpG-sites (cg07733851 and cg27122725) were identified as located in CpG shores, i.e. regions up to 2000 base pairs from CpG islands. No data suggested the differentially methylated sites were positioned in RNA polymerase-II binding sites.

These results are in line with the literature reporting that *NR3C1* methylation has also been associated with the function of the HPA axis. A previous study, evaluating salivary cortisol before, during, and after a social stress task, reported that a flattened cortisol recovery slope was associated with higher *NR3C1* methylation levels suggesting that methylation of *NR3C1* may impair negative feedback of the HPA axis^34^. In addition, another recent study reported that morning cortisol levels were positively associated to the degree of methylation of the *NR3C1* exon 1F and mean DNA methylation levels were significantly increased in depressed patients reflecting glucocorticoid receptor resistance^35^. In the same line, a study by Weder et al. (2014), reported that increased methylation of one CPG (cg04111177) of the NR3C1 gene was associated with higher morning cortisol^36^. Moreover, Stonawski et al., reported a sex difference with one CpG (cg07733851) of the *NR3C1* gene, having a main effect, hypermethylated, and two other *NR3C1* CpGs (cg04111177, cg27107893) interacting with sex in girls showing higher methylation values than boys after prenatal exposure to depressive symptoms^37^. The majority of studies investigating differential *NR3C1-1F* DNA methylation and baseline cortisol levels or after stress challenges (e.g. Trier Social Stress Test or the Dex/CRH test) reported higher cortisol stress responses as well as attenuated HPA axis feedback regulation^38–40^. In fact, childhood adversity as well as psychiatric disorders such as depression, anxiety and substance-use disorders were related with reduced methylation of *NR3C1*^40^. A recent study, reported also that the methylation of specific CG sites within the *NR3C1* exon 1F predicted a steeper diurnal cortisol slope instead of cortisol levels in the morning, afternoon or bedtime^41^.

The methylation levels of cg08636224 -associated with *FKBP5* transcript NM_004117 was significantly correlated with baseline cortisol and positively correlated with gene expression of the *FKBP5* gene in an independent cohort. However, the magnitude of methylation difference was small (1%), that might question possible biological effects. However, there is growing evidence suggesting a wide range of transcriptional and translational effects due to subtle changes in methylation on specific genes. This has been shown especially in complex, multifactorial diseases such as schizophrenia and depression^42^**.** The FK506 binding protein 5 (FKBP5) reduces the affinity of cortisol to the glucocorticoid receptor complex thus leading to the inhibition of the HPA axis ^43,44^. It has been previously reported that genetic polymorphisms are very important when investigating the function of the *FKBP5* gene especially in the context of gene x environment interaction especially childhood trauma^44^. However, a previous study in epigenetics of the *FKBP5* gene, using a targeted approach, investigated cortisol stress responses in a sample of healthy volunteers in relation to exposure to childhood trauma. The authors reported no association between *FKBP5* DNA methylation levels with both acute and chronic cortisol output nor an effect of exposure of childhood trauma and emphasize on the significance of the mental health status of the participants^45^. Thus, both methodological differences and possible confounders such as psychiatric disorders and childhood adversity are important when assessing methylation patterns of the *FKBP5* gene and the HPA axis function. In addition, results of the post hoc analysis regarding the effect of hypersexuality on methylation, indicate that the identified association between cg07733851 methylation levels and DST non-suppression status holds true irrespective of occurrence of hypersexual disorder diagnosis, but that the effect is amplified in these subjects. Underlying the strength of the present study, no other candidate CpG-site showed significant results on DNA methylation levels in dependency of hypersexual disorder nor in dependency of any interaction between DST non-suppression status and hypersexual disorder diagnosis (*P* > 0.05), indicating no bias from disease-state dependent effects on the direction of the methylation change.

Finally, the TSH/T4-ratio, as a more sensitive measurement of the thyroid function, was associated with DNA methylation values at the *CRHR2* gene (transcript EU012442). Additionally, *CRHBP* and *CRH* (cg21842274 and cg18640030) associated DNA methylation shifts were inversely and positively correlated with gene expression, respectively. Indeed, HPA and HPT axes are close related with cortisol having a direct inhibitory effect on TRH secretion and subsequently on TSH release. It was proposed that hypercortisolemia decreases TRH mRNA levels and glucocorticoid administration decreases TSH secretion as well as TRH-induced TSH stimulation^11,46^. This was also reported in patients with Cushing’s disease^47^. On the other hand, studies reported that glucocorticoids stimulate the *TRH* gene^12^ and in animals is was reported that CRH through binding to the CRH- type 2 receptors on thyrotropes leads to increased release of TSH^48,49^. Thus, it seems that CRH and glucocorticoids exhibit antagonistic effects on TSH secretion, as it is shown in the present study, and the explicit relationship between HPA and HPT axis remains unknown^13^.

The present study has a number of strengths. To our knowledge, this is the first study to assess the DNA methylation level of the HPA axis related genes in the same sample and to examine DNA methylation differences in relation to HPA axis activity through different measurements such as pre and post DST inhibition test. We used genome-wide methylation chips with over 850-KCpG sites, however, based on our earlier findings on HPA dysregulation in men with hypersexual disorder^10^, we applied a targeted approach on candidate genes of the HPA axis. Moreover, there was a rigorous characterization of the patient population with structured diagnostics of hypersexual disorder, the presence of age matched healthy control group and important confounders, such as hypersexuality, baseline cortisol levels, were taken into consideration on the association analyses between methylation of HPA axis related genes and DST non-suppression status. Antidepressant medication as well as depression severity were not significantly associated with HPA function measures in this study population^5^. Regarding childhood adversity, a known cofounder in methylation studies on HPA axis^50^, there were no differences between DST suppressors and non-suppressors. Finally, methylation levels were correlated to gene expression in two independent cohorts.

Limitations of this study include the self-reported measurements of early life adversity (CTQ) and the cross-sectional design of the study, that does not allow any conclusions about causality. Technical validation by bisulfite-sequencing (or MassARRAY) would increase the validity of the methylation results. However, the identification of *NR3C1* related transcript AJ877169 to be associated with two independent variables in separate analysis significantly strengthens the study outcome and lends considerable support to suggest the findings were not identified by chance, i. e. in relation to DST non-suppression and DST cortisol levels. In addition, supporting the potential functional significance of the identified CpG-sites, we provide evidence for a correlation with gene expression levels. We did not control for the dexamethasone plasma concentrations during the DST^51^ or other possible confounding factors with an effect on methylation patterns such as diet, prandial states and smocking^27^. However, we reduce the likelihood of confounding due to interindividual variance performing correlation analyses intra-individually. Furthermore, the association analysis of methylation and expression were significant in the robust models, but not by Pearson correlations. This could be explained by the fact that robust linear models are recommended in small sample size in order to compensate for potential outliers or heteroscedasticity in the data^52^. We used blood DNA as target tissues are not accessible. In the investigation of blood–brain correlations of the candidate CpG-sites we report transferability between blood- and brain methylation levels in the BA20 region of the brain for *NR3C1*-associated CpG-sites cg07733851 and cg27122725 and some functional relevance of the BA7-region for cg27122725. As epigenetic patterns vary from tissue to tissue and blood, further research is needed before assuredly inferring causality as to the precise effects of the observed findings in target tissues. Previous studies have also reported significant correlations between plasma and CSF Cortisol indicating that plasma Cortisol is considered a reliable proxy for CSF levels^53^. Replication of these findings in a larger sample before generalization is required. Finally, as DNA methylation and other epigenetic markers are not static but undergo dynamic changes, the interpretation of these findings should be done with caution.

## Supplementary Information

Supplementary Information.

## References

[1] Kadmiel M, Cidlowski JA (2013). Glucocorticoid receptor signaling in health and disease. Trends Pharmacol. Sci..

[2] Pariante CM, Lightman SL (2008). The HPA axis in major depression: classical theories and new developments. Trends Neurosci..

[3] Speer KE, Semple S, Naumovski N, D'Cunha NM, McKune AJ (2019). HPA axis function and diurnal cortisol in post-traumatic stress disorder: a systematic review. Neurobiol. Stress.

[4] Lovallo WR (2006). The hypothalamic-pituitary-adrenocortical axis in addiction. Int. J. Psychophysiol..

[5] Chatzittofis A (2016). HPA axis dysregulation in men with hypersexual disorder. Psychoneuroendocrinology.

[6] Zhu H, Wang G, Qian J (2016). Transcription factors as readers and effectors of DNA methylation. Nat. Rev. Genet..

[7] Wagner JR (2014). The relationship between DNA methylation, genetic and expression inter-individual variation in untransformed human fibroblasts. Genome Biol..

[8] Argentieri MA, Nagarajan S, Seddighzadeh B, Baccarelli AA, Shields AE (2017). Epigenetic pathways in human disease: the impact of DNA methylation on stress-related pathogenesis and current challenges in biomarker development. EBioMedicine.

[9] Labonté B, Azoulay N, Yerko V, Turecki G, Brunet A (2014). Epigenetic modulation of glucocorticoid receptors in posttraumatic stress disorder. Transl. Psychiatry.

[10] Jokinen J (2017). Methylation of HPA axis related genes in men with hypersexual disorder. Psychoneuroendocrinology.

[11] Brabant G (1987). Circadian and pulsatile thyrotropin secretion in euthyroid man under the influence of thyroid hormone and glucocorticoid administration. J. Clin. Endocrinol. Metab..

[12] Luo LG, Jackson IM (1998). Glucocorticoids stimulate TRH and c-fos/c-jun gene co-expression in cultured hypothalamic neurons. Brain Res..

[13] Mokrani MC, Duval F, Erb A, Gonzalez Lopera F, Danila V (2020). Are the thyroid and adrenal system alterations linked in depression?. Psychoneuroendocrinology.

[14] Kafka MP (2010). Hypersexual disorder: a proposed diagnosis for DSM-V. Arch. Sex Behav..

[15] Sheehan DV (1998). The Mini-International Neuropsychiatric Interview (M.I.N.I.): the development and validation of a structured diagnostic psychiatric interview for DSM-IV and ICD-10. J. Clin. Psychiatry.

[16] Kalichman SC, Rompa D (1995). Sexual sensation seeking and Sexual Compulsivity Scales: reliability, validity, and predicting HIV risk behavior. J. Pers. Assess.

[17] Svanborg P, Asberg M (2001). A comparison between the Beck Depression Inventory (BDI) and the self-rating version of the Montgomery Asberg Depression Rating Scale (MADRS). J. Affect. Disord..

[18] Bernstein, D. P. & Fink, L. *Childhood Trauma Questionnaire: A Retrospective Self-Report Manual*. (The Psychological Corporation, 1998).

[19] Sambrook, J., Fritsch, E.F, Maniatis, T. *Molecular Cloning A Laboratory Manual Second Edition*. Vol. 1,2,3 (Cold Spring Harbor Lab Press, 1989).

[20] Aryee MJ (2014). Minfi: a flexible and comprehensive Bioconductor package for the analysis of Infinium DNA methylation microarrays. Bioinformatics.

[21] Schalkwyk, L. *wateRmelon: Illumina 450 methylation array normalization and metrics*, https://www.bioconductor.org/packages/release/bioc/html/wateRmelon.html (2013).

[22] Leek JT, Johnson WE, Parker HS, Jaffe AE, Storey JD (2012). The sva package for removing batch effects and other unwanted variation in high-throughput experiments. Bioinformatics.

[23] Morris TJ (2013). ChAMP: 450k chip analysis methylation pipeline. Bioinformatics.

[24] Lê, S., Josse, J. & Husson, F. FactoMineR: An R Package for Multivariate Analysis. *Journal of Statistical Software* **25**, 10.18637/jss.v025.i01 (2008).

[25] Voisin S (2015). Many obesity-associated SNPs strongly associate with DNA methylation changes at proximal promoters and enhancers. Genome Med..

[26] Price ME (2013). Additional annotation enhances potential for biologically-relevant analysis of the Illumina Infinium HumanMethylation450 BeadChip array. Epigenetics Chromatin.

[27] Rask-Andersen M (2016). Postprandial alterations in whole-blood DNA methylation are mediated by changes in white blood cell composition. Am. J. Clin. Nutr..

[28] Steegenga WT (2014). Genome-wide age-related changes in DNA methylation and gene expression in human PBMCs. Age (Dordr).

[29] Du P (2010). Comparison of Beta-value and M-value methods for quantifying methylation levels by microarray analysis. BMC Bioinf..

[30] Smyth GK (2004). Linear models and empirical bayes methods for assessing differential expression in microarray experiments. Stat. Appl. Genetics Mol. Biol..

[31] Benjamini Y, Hochberg Y (1995). Controlling the false discovery rate: a practical and powerful approach to multiple testing. J. R. Stat. Soc. Ser. B Methodol..

[32] Hampel, F. R., Ronchetti, E. M., Rousseeuw, P.J., and Stahel, W.A. *Robust Statistics: The Approach Based on Influence of Functions.* (Wiley, 1986).

[33] Edgar RD, Jones MJ, Meaney MJ, Turecki G, Kobor MS (2017). BECon: a tool for interpreting DNA methylation findings from blood in the context of brain. Transl. Psychiatry.

[34] van der Knaap LJ, Oldehinkel AJ, Verhulst FC, van Oort FV, Riese H (2015). Glucocorticoid receptor gene methylation and HPA-axis regulation in adolescents. The TRAILS study. Psychoneuroendocrinology.

[35] Farrell C (2018). DNA methylation differences at the glucocorticoid receptor gene in depression are related to functional alterations in hypothalamic-pituitary-adrenal axis activity and to early life emotional abuse. Psychiatry Res..

[36] Weder N (2014). Child abuse, depression, and methylation in genes involved with stress, neural plasticity, and brain circuitry. J. Am. Acad. Child Adolesc. Psychiatry.

[37] Stonawski V (2019). Associations of prenatal depressive symptoms with DNA methylation of HPA axis-related genes and diurnal cortisol profiles in primary school-aged children. Dev. Psychopathol..

[38] Alexander N (2018). Glucocorticoid receptor gene methylation moderates the association of childhood trauma and cortisol stress reactivity. Psychoneuroendocrinology.

[39] Palma-Gudiel H, Córdova-Palomera A, Leza JC, Fañanás L (2015). Glucocorticoid receptor gene (NR3C1) methylation processes as mediators of early adversity in stress-related disorders causality: a critical review. Neurosci. Biobehav. Rev..

[40] Tyrka AR (2016). Methylation of the leukocyte glucocorticoid receptor gene promoter in adults: associations with early adversity and depressive, anxiety and substance-use disorders. Transl. Psychiatry.

[41] Lewis CR (2020). Harsh parenting predicts novel HPA receptor gene methylation and NR3C1 methylation predicts cortisol daily slope in middle childhood. Cell Mol. Neurobiol..

[42] Leenen FA, Muller CP, Turner JD (2016). DNA methylation: conducting the orchestra from exposure to phenotype?. Clin. Epigen..

[43] Liu PZ, Nusslock R (2018). How stress gets under the skin: early life adversity and glucocorticoid receptor epigenetic regulation. Curr. Genom..

[44] Zannas AS, Binder EB (2014). Gene-environment interactions at the FKBP5 locus: sensitive periods, mechanisms and pleiotropism. Genes. Brain Behav..

[45] Alexander N, Kirschbaum C, Stalder T, Muehlhan M, Vogel S (2020). No association between FKBP5 gene methylation and acute and long-term cortisol output. Transl. Psychiatry.

[46] Re RN, Kourides IA, Ridgway EC, Weintraub BD, Maloof F (1976). The effect of glucocorticoid administration on human pituitary secretion of thyrotropin and prolactin. J. Clin. Endocrinol. Metab..

[47] Roelfsema F (2009). Diminished and irregular TSH secretion with delayed acrophase in patients with Cushing's syndrome. Eur. J. Endocrinol..

[48] Geris KL, De Groef B, Kühn ER, Darras VM (2003). In vitro study of corticotropin-releasing hormone-induced thyrotropin release: ontogeny and inhibition by somatostatin. Gen. Comp. Endocrinol..

[49] De Groef B, Goris N, Arckens L, Kuhn ER, Darras VM (2003). Corticotropin-releasing hormone (CRH)-induced thyrotropin release is directly mediated through CRH receptor type 2 on thyrotropes. Endocrinology.

[50] Szyf M, Bick J (2012). DNA methylation: a mechanism for embedding early life experiences in the genome. Child Dev..

[51] Menke A (2016). Time-dependent effects of dexamethasone plasma concentrations on glucocorticoid receptor challenge tests. Psychoneuroendocrinology.

[52] Joubert BR (2012). 450K epigenome-wide scan identifies differential DNA methylation in newborns related to maternal smoking during pregnancy. Environ. Health Perspect..

[53] Chatzittofis A (2013). CSF 5-HIAA, cortisol and DHEAS levels in suicide attempters. Eur. Neuropsychopharmacol..

