# Supplementary material for: HPA axis dysregulation is associated with differential methylation of CpG-sites in related genes
Source: Sci Rep. 2021 Oct 11;11:20134. doi: 10.1038/s41598-021-99714-x (PMC8505644; doi:10.1038/s41598-021-99714-x)
Supplement: Supplementary file 1 — Supplementary Information. [file 41598_2021_99714_MOESM1_ESM.docx]

***2.3.* *Assessments***

The Wed based platform included the following self-rated scales:

*Hypersexual disorder screening inventory (HDSI)* consists of 7 items following the criteria (5A and 2B criteria) of hypersexual disorder. These are graded 0-4, from “never true” to “almost always true” during the past 6 months, total score ranges from 0-28. For a possible diagnosis of Hypersexual Disorder a minimum score of 3 is required on 4 out of 5 A-criteria, and 3 or 4 points on a minimum of 1 B-criteria is required with minimum total score of 15 (www.dsm5.org).

The *Sexual Compulsivity Scale (SCS)* is a 10-item measure where respondents endorse agreement with statements about sexually compulsive behavior, sexual preoccupations, and sexually intrusive thoughts on 4-point scales (1 “not at all like me” to 4 “very much like me”). Total score range 10-40 with < 18 classified as not sexually compulsive, 18-23 as mild sexual compulsivity, 24-29 as moderate and >30 as sexually compulsive. It was developed for assessing high-risk sexual behaviours ([Kalichman and Rompa, 1995](#_ENREF_21)).

*The Hypersexual Disorder: Current Assessment Scale (HD:CAS)* was used to assess the symptoms in the recent 2-week time and is considered to be the dimensional measurement of hypersexual behavior. The HD:CAS contains seven questions with the first one (A1) asking for the type as well as the number of sexual behaviors (includes masturbation, pornography, sex with consenting adults, cybersex, telephone sex, strip clubs, and other sexual behaviors). The following six questions (A2–A7) quantify these symptoms during the recent 2-week time frame. Each question (A2–A7) is rated in a 5 point intensity scale (0–4) with total scores of the HD:CAS between 0 to 24 points.

The *Montgomery-Åsberg Depression Rating Scale Self rating* *(MADRS-S)* was used to assess the severity of depression([Svanborg and Asberg, 2001](#_ENREF_35)). The rating scale includes nine questions on depressive symptoms, rated from 0-6 points, with total scored 0-54.

The *Childhood Trauma Questionnaire (CTQ)* was used for self-assessment of childhood trauma and includes 28 items, has five subscales, each with 5 items, measuring Emotional Abuse (EA), Physical Abuse (PA), Sexual Abuse (SA), Emotional Neglect (EN) and Physical Neglect (PN). Each subscale gets scores between 5 and 25 and classifies maltreatment as none, low, moderate and severe. The remaining 3 items constitute the minimization/denial scale used to identify individuals who may be underreporting traumatic events ([Bernstein and Fink, 1998](#_ENREF_2)). For clinical characteristics of patients with hypersexual disorder and healthy volunteers please see **Table 1.**

***2.6. Data processing***

*Background correction, adjustment of type I and type II probes, removal of batch effects and probe exclusion*

Methylation idat files were first loaded into the R environment using the ‘read.metharray.exp’ function of the minfi package. Thereafter, the efficient NOOB method was selected to correct for background artifacts (Triche et al., 2013). Probes on the Illumina Methylation EPIC BeadChip array come in two different designs which differ in dynamic range and distribution of the DNA methylation pattern. The Beta Mixture Quantile Dilation (BMIQ) function of the wateRmelon package was used to adjust the methylation data for these probe type differences (Teschendorff et al., 2013). In addition, the use of different analysis plates could result in undesired batch effects and we used the ‘ComBat’ function of the sva package to correct for this potential bias (Johnson et al., 2007). Moreover, methylation levels of CpG sites annotated to known SNP loci could be affected by single nucleotide polymorphisms (SNP:s) (Chen et al., 2013) and probes located on sex chromosomes have been shown to be more difficult to accurately normalize (Fortin et al., 2014). 196,202 CpG sites were thus subsequently excluded as they were located on sex determining chromosomes or covering known SNP loci. In addition, 2,032 probes were also filtered out as 75% or more of the samples exhibited a detection p-value >10-5. After the probe exclusion steps outlined above, 668,602 CpG sites were included in the subsequent analysis.

Correction for white blood cell type heterogeneity

DNA methylation measured in whole blood is composed of different cell populations (Reinius et al., 2012). Rask-Andersen et al. showed that changes in leukocyte fractions could introduce significant variability in the DNA methylation pattern, an effect that could bias downstream analyses. It is thus important to adjust the global DNA methylation pattern for white blood cell type heterogeneity (Rask-Andersen et al., 2016). For this purpose, we used the ‘champ.refbase’ function of the ChAMP package, which implements a statistical procedure of the Houseman algorithm to estimate the relative proportions of CD4+ and CD8+ T cells, monocytes, granulocytes, B cells and natural killer cells based on the DNA methylation pattern (Houseman et al., 2012). Using the estimated relative proportions of leukocyte subpopulations, methylation data was thereafter adjusted for white blood cell type heterogeneity in a method similar to regression calibration (‘RefBaseEWAS’) (Houseman et al., 2012).

**3. Results**

3.2. Investigation of 76 HPA axis coupled probes reveals *NR3C1* transcript AJ877169 to have a statistically significant abundance of probes differentially methylated by DST non-suppression status

**(Table 2-3, Figures 1-2)**

3.3. AJ877169 is shown to have also a statistically significant abundance of CpG sites correlated with DST cortisol levels.

**(Table 4-5)**

3.5. No HPA axis coupled transcript or individual CpG site was significantly correlated with ACTH levels, both pre and post DST

**(Table 6-7)**

3.6. Methylation levels of cg08636224 – associated with *FKBP5* transcript NM_004117 - is significantly correlated with baseline cortisol

**(Table 8)**

3.8. CpG sites associated with the genes *CRH, CRHR1, CRHR2* and *FKBP5* are significantly correlated with the TSH/T4-ratio

**(Table 9-10)**

Blood-Brain Epigenetic Concordance tool (BECon;https://redgar598.shinyapps.io/BECon/) to investigate blood-brain correlations of the candidate CpG-sites. Transferability between blood- and brain methylation levels occur specifically in the BA20 region of the brain for *NR3C1*-associated CpG-sites cg07733851 and cg27122725 In the case of cg27122725, the BA7-region appear of some functional relevance as well (**Figures 3-6** ).

These data suggest that the identified DNA methylation sites may also be differentially methylated in these brain regions.

**Table 1.** Clinical characteristics of patients with hypersexual disorder and healthy volunteers

|  | Patients  N=67 | Healthy Volunteers  N=39 | Statistics (*t-test,* Kruskall Wallis), p value |
| --- | --- | --- | --- |
|  |  |  |  |
| Age (years) Mean  Range  Std | 39.2  19-65  11.5 | 37.5  21-62  11.9 | *p=0.45* |
| Diagnosis Depression | n=11, 16.4% | - | - |
| Diagnosis Anxiety | n=12, 17.9% | - | - |
| Diagnosis other | n= 1, (ADHD) | - | - |
| Antidepressants | n=11, 16.4% | - | - |
| HDSI Mean  Range  Std | 19.6  6-28  5.7 | 1.6  0-9  2.2 | p<0.001 |
| SCS Mean  Range  Std | 27.8  12-39  6.9 | 11.1  10-14  1.2 | p<0.001 |
| HD:CAS Mean  Range  Std | 10.3  1-22  5.4 | 0.38  0-4  0.88 | p<0.001 |
| MADRS Mean  Range  Std | 18.9  1-50  9.7 | 2.4  0-12  2.9 | p<0.001 |
| CTQ Total (n=65) Mean  Range  Std | 39.95  25-80  11.48 | 32.53  25-70  8.75 | p<0.001 |

**Table 2.** Methylation changes in HPA-axis coupled CpG sites associated with DST non-suppression status

| **Gene** | **Transcript** | | **Illumina ID** | **logFC** | ***p*** | ***p* (FDR)** |
| --- | --- | --- | --- | --- | --- | --- |
| ***NR3C1*** | **AJ877169** |  | **cg07733851** | **0.08** | **1.73E-03** | ***ns*** |
| *CRHR1* | BC037967 |  | cg12577105 | 0.03 | **3.09E-03** | *ns* |
| ***NR3C1*** | **AJ877169** |  | **cg27122725** | **0.04** | **1.33E-02** | ***ns*** |
| *CRHR2* | EU012442 |  | cg13094036 | 0.00 | **1.90E-02** | *ns* |
| *CRH* | NM_000756 | | cg00468228 | 0.05 | **4.60E-02** | *ns* |
| *FKBP5* | NM_004117 | | cg20813374 | 0.04 | **4.87E-02** | *ns* |
| *CRHR2* | EU012442 |  | cg03667083 | 0.02 | 5.35E-02 | *ns* |
| *CRHR1* | AK297590 |  | cg15413793 | -0.02 | 8.48E-02 | *ns* |
| *CRHR2* | EU012442 |  | cg21773872 | -0.01 | 9.14E-02 | *ns* |
| *NR3C1* | AK302050 |  | cg18849621 | 0.01 | 9.32E-02 | *ns* |
| Analysis by multiple linear regression models of methylation M-values to a binary outcome variable of DST non-suppression (Y/N), adjusting for hypersexuality disorder (Y/N) and baseline Cortisol (nmol/L). 76 CpG-sites were analysed, located within 2000 bp of the transcriptional start site of known HPA-axis genes CRH, CRHR1, CRHR2, CHRBP, NR3C1 and FKBP5. P-values were corrected for multiple-testing using the false discovery rate (FDR)-method. Abbreviations: logFC, log fold change; p, p-value; p (FDR), FDR-adjusted p-value | | | | | | |

**Table 3.** Investigation of transcripts by binomial testing of the number of differentially methylated CpG sites by DST non-suppresion status

|  |  | **CpG-sites** | | | |
| --- | --- | --- | --- | --- | --- |
|  |  |  |  |  |  |
| **Gene** | **Transcript** | **Total** | **Sign.^a^** | ***p* (binomial)^b^** | ***p* (binomial, bonf.)^c^** |
| ***NR3C1*** | **AJ877169** | **2** | **2** | **2.50E-03** | **4.75E-02** |
| *CRHR1* | BC037967 | 3 | 1 | *ns* | *ns* |
| *FKBP5* | NM_004117 | 6 | 1 | *ns* | *ns* |
| *CRH* | NM_000756 | 6 | 1 | *ns* | *ns* |
| *CRHR2* | EU012442 | 14 | 1 | *ns* | *ns* |
| *CRHR1* | AF369651 | 1 | 0 | *ns* | *ns* |
| *FKBP5* | NM_001145776 | 3 | 0 | *ns* | *ns* |
| *CRH* | CCDS6188 | 4 | 0 | *ns* | *ns* |
| *FKBP5* | NM_001145775 | 7 | 0 | *ns* | *ns* |
| *NR3C1* | NM_001204264 | 3 | 0 | *ns* | *ns* |
| *CRHBP* | AK311052 | 4 | 0 | *ns* | *ns* |
| *CRHR2* | NM_001202481 | 2 | 0 | *ns* | *ns* |
| *CRHR1* | EU012435 | 9 | 0 | *ns* | *ns* |
| *NR3C1* | HQ450644 | 1 | 0 | *ns* | *ns* |
| *NR3C1* | AK302050 | 6 | 0 | *ns* | *ns* |
| *CRHR1* | AK297590 | 2 | 0 | *ns* | *ns* |
| *NR3C1* | AJ877168 | 1 | 0 | *ns* | *ns* |
| *NR3C1* | NM_001204265 | 1 | 0 | *ns* | *ns* |
| *CRHBP* | NM_001882 | 1 | 0 | *ns* | *ns* |
| ^a^Number of significantly hyper- or hypomethylated CpG sites per transcript. ^b^Shown are p-values for binomial tests, performed separately for each transcript, contrasting the total number of CpG-sites per transcript to the number of nominally significant CpG-sites for each transcript. ^c^P-values adjusted for multiple testing using the Bonferroni-method | | | | | |

**Table 4.** Methylation changes in HPA-axis coupled CpG sites associated with DST cortisol levels

| **Gene** | **Transcript** | | **Illumina ID** | **logFC** | ***p*** | ***p* (FDR)** |
| --- | --- | --- | --- | --- | --- | --- |
| *FKBP5* | NM_001145775 | | cg19226017 | -1.81E-04 | **1.68E-02** | *ns* |
| ***NR3C1*** | **AJ877169** |  | **cg27122725** | **1.73E-04** | **1.89E-02** | *ns* |
| *CRH* | NM_000756 | | cg23409074 | -1.73E-04 | **2.06E-02** | *ns* |
| *NR3C1* | NM_001204265 | | cg18146873 | -7.44E-05 | **2.86E-02** | *ns* |
| ***NR3C1*** | **AJ877169** |  | **cg07733851** | **2.17E-04** | **3.16E-02** | *ns* |
| *CRHR1* | EU012435 |  | cg11338426 | -2.91E-05 | **3.23E-02** | *ns* |
| *CRHR1* | EU012435 |  | cg24353392 | 1.35E-04 | **4.00E-02** | *ns* |
| *CRHR2* | EU012442 |  | cg13094036 | -1.76E-05 | **4.03E-02** | *ns* |
| *CRHR2* | EU012442 |  | cg04863452 | -5.19E-05 | **4.39E-02** | *ns* |
| *CRHR1* | EU012435 |  | cg24063856 | 2.35E-04 | **4.66E-02** | *ns* |
| Analysis by multiple linear regression models of methylation M-values to DST cortisol levels (nmol/L). 76 CpG-sites were analysed, located within 2000 bp of the transcriptional start site of known HPA-axis genes CRH, CRHR1, CRHR2, CHRBP, NR3C1 and FKBP5. P-values were corrected for multiple-testing using the false discovery rate (FDR)-method. Abbreviations: logFC, log fold change; p, p-value; p (FDR), FDR-adjusted p-value | | | | | | |

**Table 5.** Investigation of transcripts by binomial testing of the number of differentially methylated CpG sites correlated with DST cortisol

|  |  | **CpG-sites** | | | |
| --- | --- | --- | --- | --- | --- |
|  |  |  |  |  |  |
| **Gene** | **Transcript** | **Total** | **Sign.^a^** | ***p* (binomial)^b^** | ***p* (binomial, bonf.)^c^** |
| *CRHR1* | AF369651 | 1 | 0 | ns | ns |
| *FKBP5* | NM_004117 | 6 | 0 | ns | ns |
| *FKBP5* | NM_001145776 | 3 | 0 | ns | ns |
| *CRH* | NM_000756 | 6 | 1 | ns | ns |
| *CRH* | CCDS6188 | 4 | 0 | ns | ns |
| *FKBP5* | NM_001145775 | 7 | 1 | ns | ns |
| *NR3C1* | NM_001204264 | 3 | 0 | ns | ns |
| *CRHBP* | AK311052 | 4 | 0 | ns | ns |
| *CRHR2* | EU012442 | 14 | 2 | ns | ns |
| *CRHR2* | NM_001202481 | 2 | 0 | ns | ns |
| *CRHR1* | EU012435 | 9 | 3 | **8.36E-03** | *ns* |
| ***NR3C1*** | **AJ877169** | **2** | **2** | **2.50E-03** | **4.75E-02** |
| *NR3C1* | HQ450644 | 1 | 0 | ns | *ns* |
| *CRHR1* | BC037967 | 3 | 0 | ns | *ns* |
| *NR3C1* | AK302050 | 6 | 0 | ns | *ns* |
| *CRHR1* | AK297590 | 2 | 0 | ns | *ns* |
| *NR3C1* | AJ877168 | 1 | 0 | ns | *ns* |
| *NR3C1* | NM_001204265 | 1 | 1 | **5.00E-02** | *ns* |
| *CRHBP* | NM_001882 | 1 | 0 | ns | *ns* |
| ^a^Number of significantly hyper- or hypomethylated CpG sites per transcript. ^b^Shown are p-values for binomial tests, performed separately for each transcript, contrasting the total number of CpG-sites per transcript to the number of nominally significant CpG-sites for each transcript. ^c^P-values adjusted for multiple testing using the bonferroni-method | | | | | |

**Table 6.** Methylation changes in HPA-axis coupled CpG sites associated with ACTH levels

| **Gene** | **Transcript** | | **Illumina ID** | **logFC** | ***p*** | ***p* (FDR)** |
| --- | --- | --- | --- | --- | --- | --- |
| *NR3C1* | AK302050 |  | cg10847032 | **-1.74E-03** | **7.04E-03** | *ns* |
| *CRH* | NM_000756 | | cg16664570 | **-7.42E-03** | **2.10E-02** | *ns* |
| *CRHR1* | EU012435 |  | cg11731737 | **1.55E-03** | **3.14E-02** | *ns* |
| *CRH* | CCDS6188 | | cg15971888 | 9.76E-04 | 5.30E-02 | *ns* |
| *FKBP5* | NM_004117 | | cg20813374 | -4.47E-03 | 8.81E-02 | *ns* |
| *CRHR2* | EU012442 |  | cg07658503 | -1.60E-03 | 8.92E-02 | *ns* |
| *NR3C1* | AJ877169 |  | cg07733851 | -4.66E-03 | 9.23E-02 | *ns* |
| *FKBP5* | NM_001145776 | | cg00862770 | - | *ns* | *ns* |
| *FKBP5* | NM_001145775 | | cg07485685 | - | *ns* | *ns* |
| *FKBP5* | NM_001145776 | | cg00140191 | - | *ns* | *ns* |
| Analysis by multiple linear regression models of methylation M-values to baseline ACTH (pmol/L). 76 CpG-sites were analysed, located within 2000 bp of the transcriptional start site of known HPA-axis genes CRH, CRHR1, CRHR2, CHRBP, NR3C1 and FKBP5. P-values were corrected for multiple-testing using the false discovery rate (FDR)-method. Abbreviations: logFC, log fold change; p, p-value; p (FDR), FDR-adjusted p-value | | | | | | |

**Table 7.** Methylation changes in HPA-axis coupled CpG sites associated with DST ACTH levels

| **Gene** | **Transcript** | **Illumina ID** | **logFC** | ***p*** | ***p* (FDR)** |
| --- | --- | --- | --- | --- | --- |
| *CRHR1* | EU012435 | cg24353392 | 9.33E-03 | **1.31E-02** | *ns* |
| *NR3C1* | AJ877169 | cg07733851 | 1.39E-02 | **3.36E-02** | *ns* |
| *CRH* | NM_000756 | cg16664570 | -1.54E-02 | **3.40E-02** | *ns* |
| *CRHBP* | AK311052 | cg13777717 | 5.41E-03 | **3.69E-02** | *ns* |
| *NR3C1* | NM_001204265 | cg18146873 | -4.28E-03 | **3.91E-02** | *ns* |
| *CRHR1* | BC037967 | cg12577105 | 7.96E-03 | **4.47E-02** | *ns* |
| *NR3C1* | HQ450644 | cg08845721 | -7.77E-03 | **4.59E-02** | *ns* |
| *FKBP5* | NM_001145775 | cg19226017 | -8.54E-03 | 6.48E-02 | *ns* |
| *FKBP5* | NM_004117 | cg01294490 | -2.71E-03 | 7.64E-02 | *ns* |
| *CRHBP* | AK311052 | cg17448335 | -1.50E-03 | ns | *ns* |
| Analysis by multiple linear regression models of methylation M-values to DST ACTH (pmol/L). 76 CpG-sites were analysed, located within 2000 bp of the transcriptional start site of known HPA-axis genes CRH, CRHR1, CRHR2, CHRBP, NR3C1 and FKBP5. P-values were corrected for multiple-testing using the false discovery rate (FDR)-method. Abbreviations: logFC, log fold change; p, p-value; p (FDR), FDR-adjusted p-value | | | | | |

**Table 8.** Methylation changes in HPA-axis coupled CpG sites associated with baseline cortisol levels

| **Gene** | **Transcript** |  | **Illumina ID** | **logFC** | ***p*** | ***p* (FDR)** |
| --- | --- | --- | --- | --- | --- | --- |
| ***FKBP5*** | **NM_004117** |  | **cg08636224** | **-9.54E-05** | **3.54E-04** | **2.69E-02** |
| *NR3C1* | AJ877169 |  | cg07733851 | -1.19E-04 | 3.73E-02 | *ns* |
| *CRHR2* | EU012442 |  | cg23185751 | -8.76E-05 | 3.77E-02 | *ns* |
| *CRH* | CCDS6188 |  | cg20329958 | 9.52E-05 | 5.56E-02 | *ns* |
| *NR3C1* | AK302050 |  | cg10847032 | -2.85E-05 | 5.76E-02 | *ns* |
| *CRH* | NM_000756 |  | cg16664570 | -1.29E-04 | 6.84E-02 | *ns* |
| *CRHR2* | EU012442 |  | cg04923928 | -4.15E-05 | 7.30E-02 | *ns* |
| *NR3C1* | AJ877168 |  | cg15910486 | -2.61E-05 | 7.33E-02 | *ns* |
| *FKBP5* | NM_004117 |  | cg07843056 | -1.41E-05 | 8.69E-02 | *ns* |
| *NR3C1* | AK302050 |  | cg13648501 | 5.83E-05 | 9.53E-02 | *ns* |
| Analysis by multiple linear regression models of methylation M-values to baseline cortisol (nmol/L). 76 CpG-sites were analysed, located within 2000 bp of the transcriptional start site of known HPA-axis genes CRH, CRHR1, CRHR2, CHRBP, NR3C1 and FKBP5. P-values were corrected for multiple-testing using the false discovery rate (FDR)-method. Abbreviations: logFC, log fold change; p, p-value; p (FDR), FDR-adjusted p-value | | | | | | |

**Table 9.** TSH/T4 (mE/nmol) associated methylation changes in HPA-axis coupled CpG sites

| **Gene** | **Transcript** |  | **Illumina ID** |  | **logFC** | ***p*** | ***p* (FDR)** |
| --- | --- | --- | --- | --- | --- | --- | --- |
| *CRHR2* | EU012442 |  | cg09516959 |  | 1.49 | **4.13E-10** | **3.14E-08** |
| *FKBP5* | NM_004117 |  | cg20813374 |  | -1.57 | **3.89E-06** | **1.48E-04** |
| *CRHR1* | EU012435 |  | cg24394631 |  | 2.00 | **2.18E-05** | **5.52E-04** |
| *CRHR2* | EU012442 |  | cg21773872 |  | 0.29 | **4.45E-05** | **7.91E-04** |
| *CRHBP* | NM_001882 |  | cg21842274 |  | 1.49 | **5.20E-05** | **7.91E-04** |
| *CRHR2* | EU012442 |  | cg01718447 |  | 0.15 | **1.43E-03** | **1.81E-02** |
| *CRHR1* | EU012435 |  | cg11731737 |  | 0.26 | **4.78E-03** | **4.54E-02** |
| *CRH* | NM_000756 |  | cg18640030 |  | 1.37 | **5.93E-03** | **4.96E-02** |
| *CRHR2* | EU012442 |  | cg04922810 |  | 0.94 | **6.53E-03** | **4.96E-02** |
| *CRHR2* | EU012442 |  | cg03667083 |  | 0.79 | **7.56E-03** | 5.22E-02 |
| Analysis by multiple linear regression models of methylation M-values to TSH/T4-ratio (mE/nmol), adjusting for hypersexuality (Y/N) and IL-6 levels (ng/L). 76 CpG-sites were analysed, located within 2000 bp of the transcriptional start site of known HPA-axis genes CRH, CRHR1, CRHR2, CHRBP, NR3C1 and FKBP5. P-values were corrected for multiple-testing using the false discovery rate (FDR)-method. Abbreviations: logFC, log fold change; p, p-value; p (FDR), FDR-adjusted p-value | | | | | | | |

**Table 10.** Investigation of transcripts by binomial testing of the number of CpG sites correlated with TSH/T4 (mE/nmoL)

| **Gene** | **Transcript** | **Total** | **Sign.^a^** | ***p* (binomial)^b^** | ***p* (binomial, bonf.)^c^** |
| --- | --- | --- | --- | --- | --- |
| *CRHR1* | AF369651 | 1 | 0 | *ns* | *ns* |
| *FKBP5* | NM_004117 | 6 | 2 | **3.28E-02** | *ns* |
| *FKBP5* | NM_001145776 | 3 | 0 | *ns* | *ns* |
| *CRH* | NM_000756 | 6 | 1 | *ns* | *ns* |
| *CRH* | CCDS6188 | 4 | 1 | *ns* | *ns* |
| *FKBP5* | NM_001145775 | 7 | 0 | *ns* | *ns* |
| *NR3C1* | NM_001204264 | 3 | 0 | *ns* | *ns* |
| *CRHBP* | AK311052 | 4 | 0 | *ns* | *ns* |
| ***CRHR2*** | **EU012442** | **14** | **5** | **4.27E-04** | **8.12E-03** |
| *CRHR2* | NM_001202481 | 2 | 1 | 9.75E-02 | *ns* |
| *CRHR1* | EU012435 | 9 | 2 | 7.12E-02 | *ns* |
| *NR3C1* | AJ877169 | 2 | 1 | 9.75E-02 | *ns* |
| *NR3C1* | HQ450644 | 1 | 0 | *ns* | *ns* |
| *CRHR1* | BC037967 | 3 | 0 | *ns* | *ns* |
| *NR3C1* | AK302050 | 6 | 0 | *ns* | *ns* |
| *CRHR1* | AK297590 | 2 | 0 | *ns* | *ns* |
| *NR3C1* | AJ877168 | 1 | 1 | 5.00E-02 | *ns* |
| *NR3C1* | NM_001204265 | 1 | 0 | *ns* | *ns* |
| *CRHBP* | NM_001882 | 1 | 1 | **5.00E-02** | *ns* |
| ^a^Number of nominally significantly hyper- or hypomethylated CpG sites per transcript (p<0.05) ^b^Shown are p-values for binomial tests, performed separately for each transcript, contrasting the total number of CpG-sites per transcript to the number of nominally significant CpG-sites for each transcript. ^c^P-values adjusted for multiple testing using the Bonferroni-method | | | | | |

**Figure 1.** Percent methylation of NR3C1 associated CpG site cg07733851 by DST non-suppression status


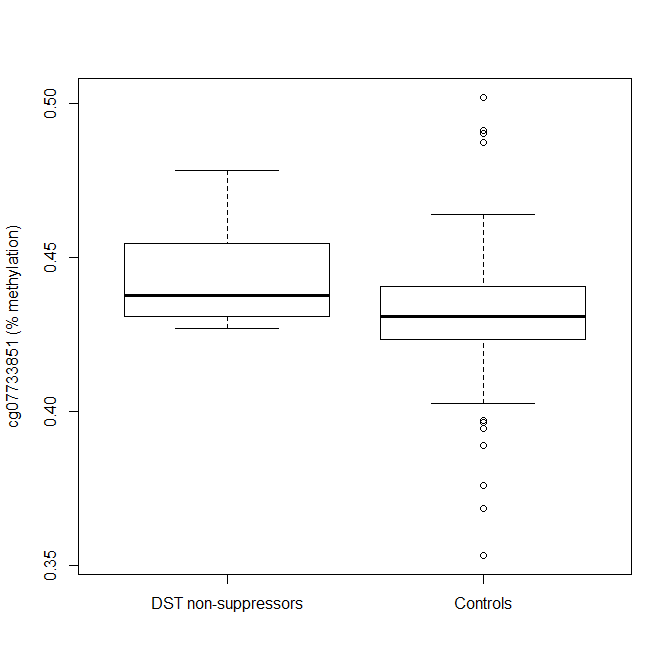


**Figure 2.** Percent methylation of *NR3C1* associated CpG site cg27122725 by DST non-suppression status


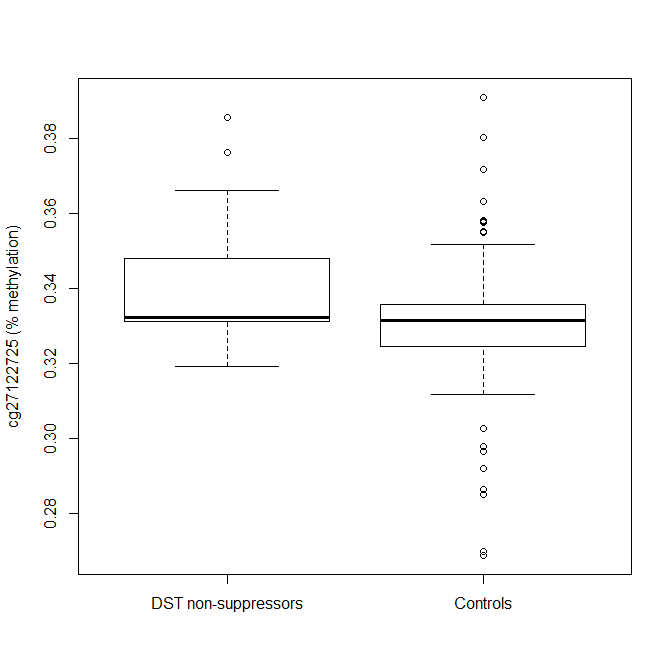


**Figures 3:** Blood-brain correlation plots *FKBP5* vs cg08636224 (BECon)


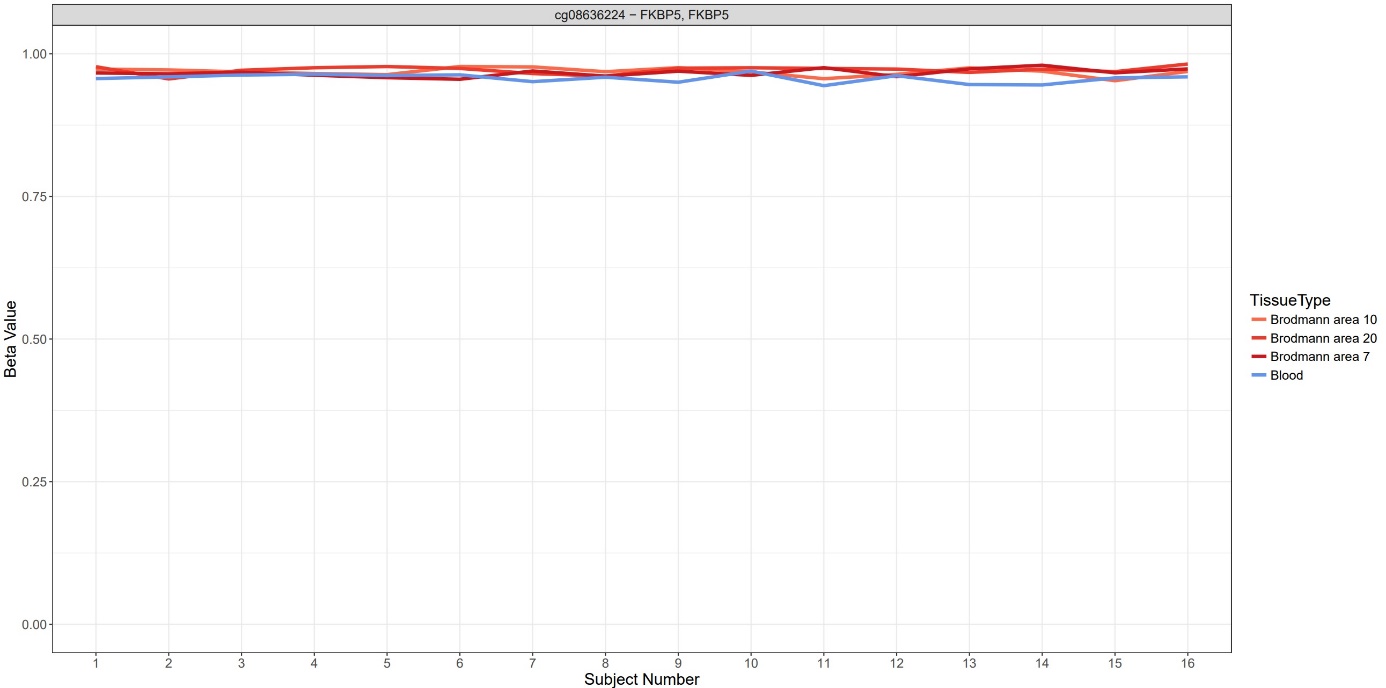


**Figure 4:** Blood-brain correlation plots *NR3c1* vs cg07733851 and cg27122725 (BECon)


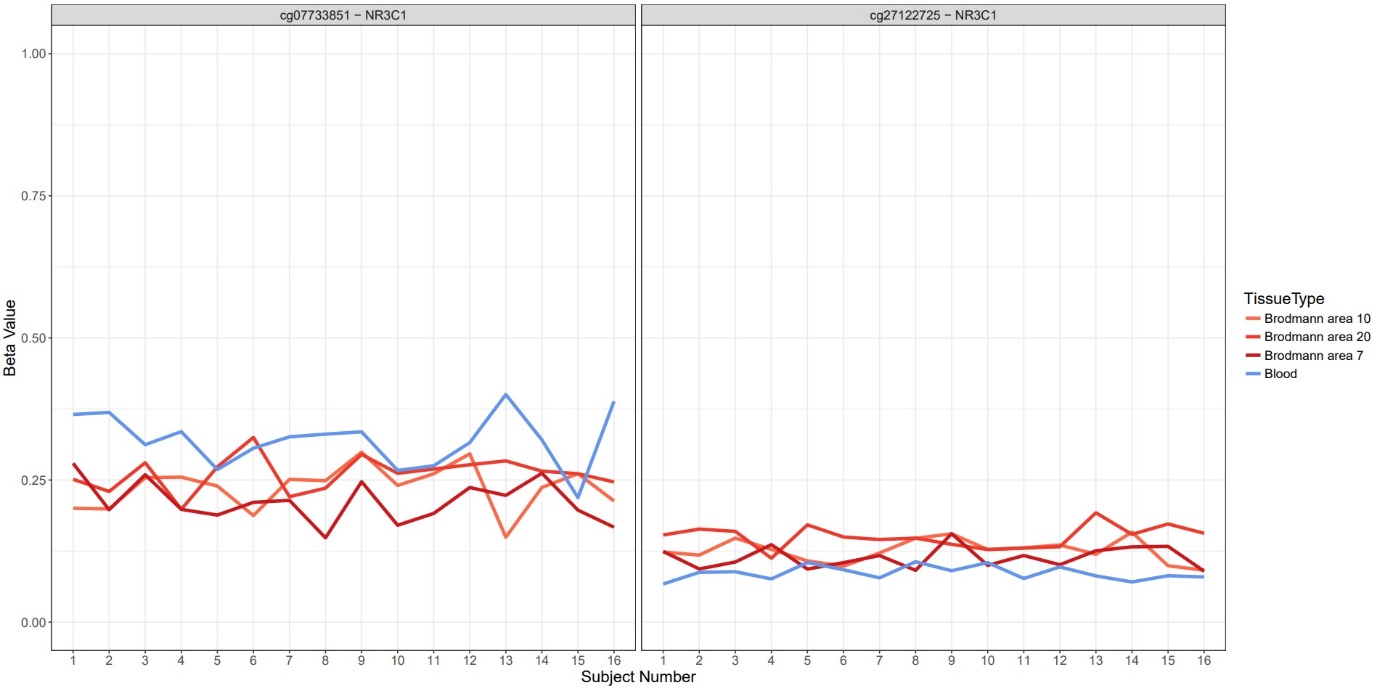


**Figure 5:** Summary of correlations and variability of cg07733851 and cg27122725 (BECon)


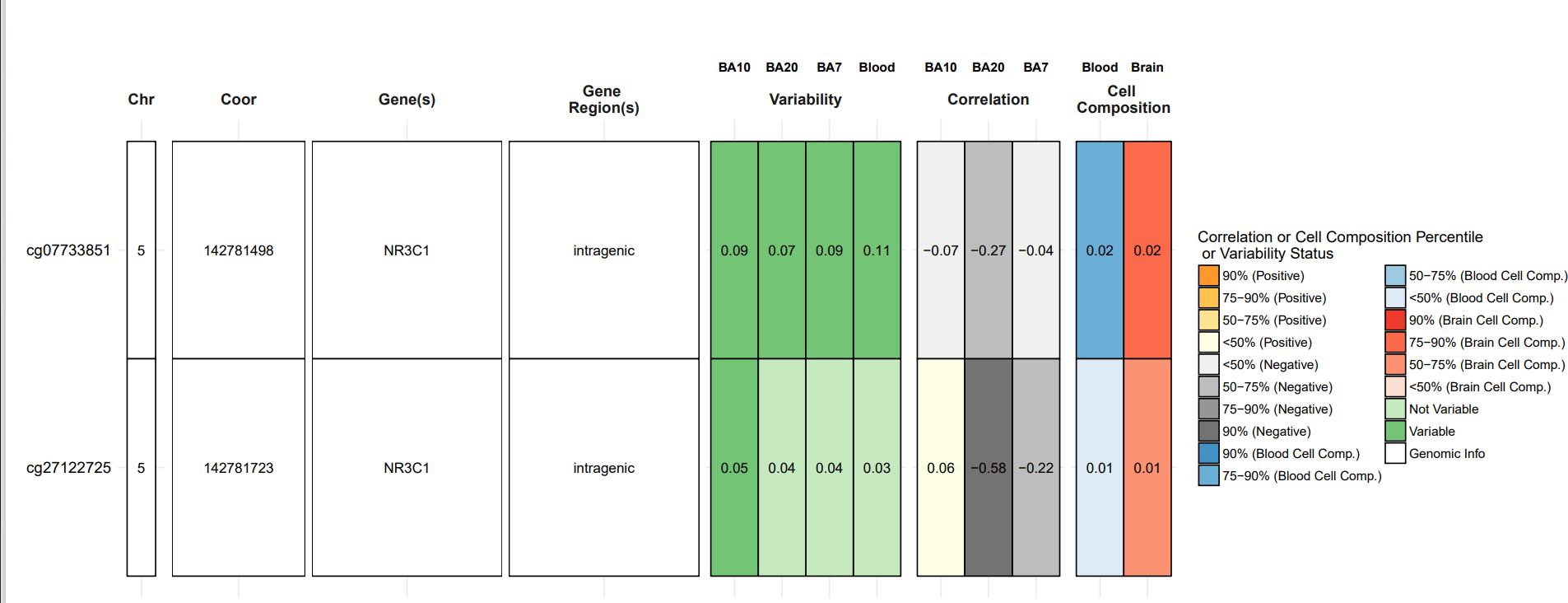


**Figure 6:** Summary of correlations and variability of cg08636224 (BECon)


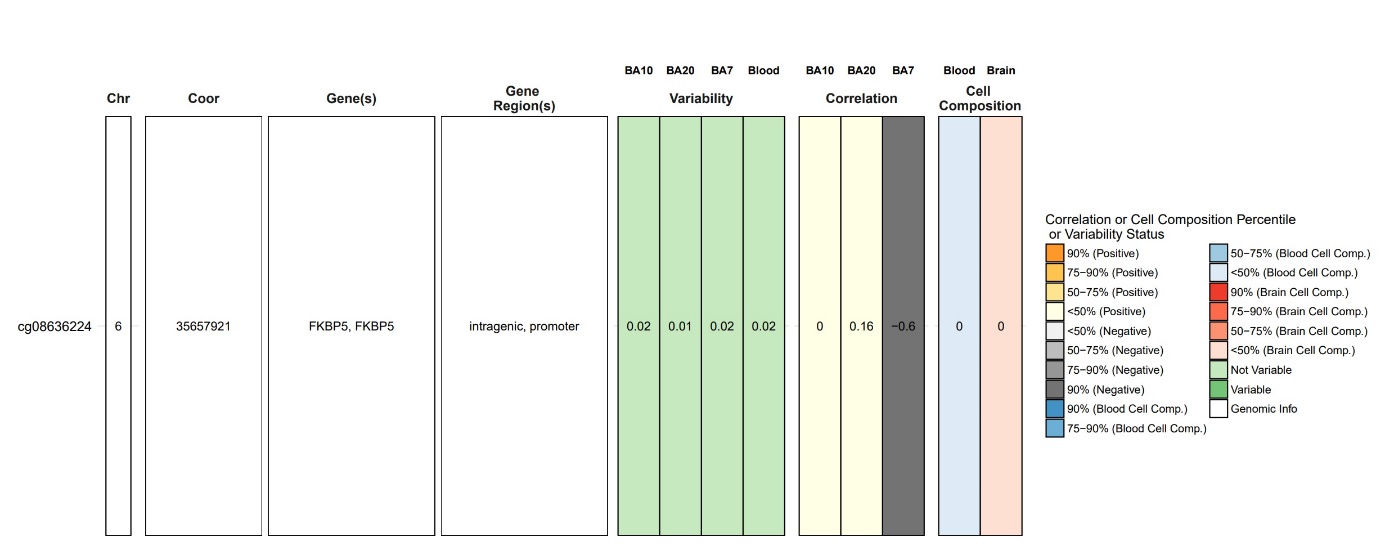


**References**

Chen, Y-a., Lemire, M., Choufani, S., Butcher, D.T., Grafodatskaya, D., Zanke, B.W., Gallinger, S., Hudson, T.J., Weksberg, R., 2013. Discovery of cross-reactive probes and polymorphic CpGs in the Illumina Infinium HumanMethylation450 microarray. Epigenetics. 2013;8(2):203-9.

Fortin, J.P., Labbe, A., Lemire, M., Zanke, B.W., Hudson, T.J., Fertig, E.J., Greenwood, C.M., Hansen, K.D., 2014. Functional normalization of 450k methylation array data improves replication in large cancer studies. Genome biology 15(12):503.

Houseman, E.A., Accomando, W.P., Koestler, D.C., Christensen, B.C., Marsit, C.J., Nelson, H.H., Wiencke, J.K., Kelsey, K.T. 2012. DNA methylation arrays as surrogate measures of cell mixture distribution. BMC Bioinformatics 13: 86.

Johnson, W.E., Li, C., Rabinovic, A., 2007. Adjusting batch effects in microarray expression data using empirical Bayes methods. Biostatistics (Oxford, England). 8(1):118-27.

Reinius, L.E., Acevedo, N., Joerink, M., Pershagen, G., Dahlen, S.E., Greco, D., Söderhäll, C., Scheynius, A., Kere, J., 2012. Differential DNA methylation in purified human blood cells: implications focr cell lineage and studies on disease susceptibility. PLoS One. 7(7): e41361.

Teschendorff, A.E., Marabita, F., Lechner, M., Bartlett, T., Tegner, J., Gomez-Cabrero, D., Beck, S., 2012. A Beta-Mixture Quantile Normalisation method for correcting probe design bias in Illumina Infinium 450k DNA methylation data. Bioinformatics. 29(2), 189-96.

Triche, T.J., Jr., Weisenberger, D.J, Van Den Berg, D., Laird, P.W., Siegmund, K.D., 2013. Low-level processing of Illumina Infinium DNA Methylation BeadArrays. Nucleic acids research. 41(7), e90.
